# Association between gut microbiota composition and ischemic colitis: a comparative analysis

**DOI:** 10.3389/fmicb.2026.1746217

**Published:** 2026-04-10

**Authors:** Rui Feng, Qi Tang, Liang Xu, Yue Yao, Hai-Yan Shen, Xin-Feng Shen, Shui-Liang Ruan

**Affiliations:** 1 Zhejiang Chinese Medical University, Hangzhou, China; 2 Department of Gastroenterology, The Second Affiliated Hospital of Jiaxing University, Jiaxing, China

**Keywords:** Firmicutes, gut microbiota, ischemic colitis, polymorphism, Proteobacteria

## Abstract

**Objective:**

This study aimed to examine the association between gut microbiota composition and ischemic colitis (IC) by comparing the microbial diversity and abundance in individuals diagnosed with IC and healthy controls.

**Methods:**

A comparative analysis of gut microbiota was conducted in 18 individuals with IC and 11 healthy controls. Microbial community structure was assessed at both the phylum and genus levels.

**Results:**

At the phylum level, individuals with IC exhibited a predominance of Proteobacteria, Bacteroidetes, and Firmicutes, whereas the control group primarily harbored Phylum Firmicutes, Proteobacteria, and Actinobacteria. At the genus level, the IC group showed enrichment in *Bacteroides*, *Halomonas*, and *Escherichia-Shigella* while the control group primarily included *Akkermansia*, *Bacteroides*, and *Bifidobacterium*. Beta diversity analysis demonstrated significantly greater operational taxonomic unit richness and community diversity in the IC group compared to controls. Analysis of similarities demonstrated significant inter-group differences in microbial community composition (*r* = 0.223, *p* = 0.002). Analysis of molecular variance analysis further confirmed significant differences in microbial abundance and diversity between groups (*p* < 0.05). Linear discriminant analysis effect size identified a significant enrichment of Proteobacteria in the IC group, particularly Gammaproteobacteria, Pseudomonadales, Moraxellaceae, and *Enhydrobacter*. In contrast, the control group exhibited significant enrichment in Firmicutes, notably *Rothia*, Peptostreptococcaceae, *Clostridium sensu stricto*, Clostridiaceae, and Erysipelotrichales.

**Conclusion:**

The gut microbiota of individuals with IC was characterized by increased microbial diversity and notable alterations in community composition. Specifically, there was an enrichment of Proteobacteria and several potentially pathogenic taxa, including the genera *Bacteroides*, *Halomonas*, and *Escherichia-Shigella*, as well as members of the order Pseudomonadales. Meanwhile, we observed reductions in Firmicutes, Actinobacteria, and health-associated genera, including *Akkermansia*, *Bifidobacterium*, and *Rothia*. These microbial shifts are associated with IC, which may be related to the disruption of gut homeostasis, promotion of inflammation, and impairment of mucosal barrier function. Due to the observational and cross-sectional nature of this study, no causal relationship between microbial changes and IC can be established, and further research is needed to explore the specific mechanisms involved.

## 1 Introduction

Ischemic colitis (IC) is characterized by ischemic injury to the intestinal mucosa resulting from non-occlusive damage to the microvasculature supplying the colon. Several risk factors are associated with IC such as advancing age, sex, hypertension, diabetes mellitus, and chronic constipation. With an aging population in China and the increasing use of colonoscopy, the number of IC cases has risen (Ruan et al., 2015). However, the underlying etiology and pathogenesis of IC remain poorly understood. Recent studies have documented alterations in gut microbiota diversity and abundance in various gastrointestinal inflammatory disorders; however research focusing on gut microbial changes in IC are limited (Shen et al., 2016). Therefore, this study aims to explore the potential association between IC and gut microbiota, with an emphasis on microbial community alterations.

## 2 Data and methods

### 2.1 General information

Study group (Group A): Inclusion criteria for individuals with IC were as follows: ① Presentation at The Second Hospital of Jiaxing between January 2021 and June 2023; ② Age between 14 and 90 years and residing within the Jiaxing city region; and ③ Fulfilment of the diagnostic criteria for IC (Editorial Board of Chinese Journal of Geriatric Medicine, 2011), willingness to participate in screening and follow-up, and provision of signed informed consent.

Exclusion criteria included a diagnosis of inflammatory bowel disease, infectious enteritis, intestinal obstruction, recent use of antibiotics, or the presence of active malignancy.

A total of 18 individuals with IC were included in the study (7 males and 11 females), with an age range of 14 to 86 years and a mean age of 61.50 ± 16.61 years. Among them, 11 had hypertension, 9 had dyslipidemia and fatty liver, 7 had atherosclerosis, and 1 had diabetes mellitus.

*Control Group (Group B)*: Inclusion criteria for the control group were as follows: ① Participation in routine health examinations at the Second Hospital of Jiaxing during the same study period; ② Match with the study group in terms of age, sex, body weight, dietary habits, and long-term residency in the Jiaxing region; ③ Absence of inflammatory bowel disease, infectious bowel disease, intestinal obstruction, recent use of antibiotics, or current malignancy; ④ Willingness to participate in screening and provision of signed informed consent. A total of 11 individuals were included in the control group, (4 males and 7 females), aged 24 to 81 years, with a mean age of 62.34 ± 12.64 years. Among them, 6 had hypertension, 4 had dyslipidemia and fatty liver, 3 had atherosclerosis, and 2 had diabetes mellitus. No statistically significant differences were observed between the two groups with respect to sex or age.

*Note*: The control group finally included 11 subjects, and the IC group included 18 subjects. The sample size did not reach a 1:1 balance. The main reason is that during the study period (January 2021–June 2023), the number of volunteers who underwent physical examinations at The Second Hospital of Jiaxing and met the inclusion and exclusion criteria (no intestinal diseases, no recent use of antibiotics, long-term residence in Jiaxing, etc.) was limited. At the same time, to ensure the matching of baseline characteristics such as age, sex, and dietary habits between the two groups, some healthy examinees who did not meet the matching conditions were not included, resulting in a slightly smaller sample size in the control group.

### 2.2 Methods

#### 2.2.1 Sample collection and processing

Fresh fecal samples were obtained from individuals with suspected IC within 24 h of symptom onset. Samples were placed in 25 mL cryogenic tubes and immediately stored at −80 °C. Participants who underwent colonoscopy within 48 h of symptom onset and received a confirmed diagnosis of IC were enrolled in the study; their previously collected samples remained stored under frozen conditions. Fecal samples from individuals in the control group were collected using the same protocol and likewise stored at −80 °C immediately after collection.

#### 2.2.2 Experimental procedures

All experimental procedures were conducted at the Laboratory of Hangzhou Woosen Biotechnology (WS-Bio).

(1) DNA extraction

Following thawing, total DNA was extracted from fecal samples using a commercial extraction kit. The concentration of extracted DNA was measured, and electrophoresis was performed to evaluate DNA integrity and quality.

(2) PCR amplification

The forward (F) and reverse (R) primers targeting the V3-V4 hypervariable region of the 16S rRNA gene were diluted to 0.25 μL, and the DNA was diluted to a concentration of 10–50 ng/μL. The primers and DNA were then combined to form a 25 μL reaction mixture, which was then transferred into PCR tubes. Thermal cycling was performed under the following program: pre-denaturation, denaturation, annealing, extension, final extension, and hold. Post-amplification, 2 μL of the PCR product was subjected to electrophoresis on a 2% agarose gel to verify the presence of the target fragment. The amplified product was subsequently purified using a nucleic acid purification kit (1× volume). A second round of PCR amplification was performed using the purified product, followed by another electrophoresis step on a 2% agarose gel. The target fragment was excised and recovered using a gel extraction kit.

(3) Library quality control

DNA concentration and purity of the sequencing library were evaluated using a fluorometer. A bioanalyzer was employed to assess fragment length distribution, and a library quantification kit was used to determine the molar concentration, which served as the basis for library pooling.

(4) Sequencing

The pooled libraries were denatured and loaded onto the Illumina NovaSeq 6,000 sequencing platform for high-throughput paired-end sequencing (PE250), in which both ends of each DNA fragment were sequenced. Each sample yielded two data files: reads1 (R1) and reads2 (R2).

(4) Data processing and analysis

Raw sequencing data were subjected to quality control and filtering using the Illumina NovaSeq 6000 platform. The detailed bioinformatics pipeline was as follows: ① Sequence assembly: Paired-end reads were merged using the fastq_mergepairs command in VSEARCH; ② Primer removal: Primers were trimmed using cutadapt software; ③ Quality filtering: Low-quality sequences (containing N bases or shorter than 100 bp) were removed using the fastq_filter command in VSEARCH; ④ Chimera removal: *De novo* chimera detection and removal were performed using the uchime3_denovo command in VSEARCH, followed by reference-based chimera filtering with the uchime_ref command; ⑤ OTU clustering: Sequences with ≥97% similarity were clustered into operational taxonomic units (OTUs) using VSEARCH; ⑥ Taxonomic annotation: OTUs were annotated against the SILVA database. The processed data were then used for downstream analyses, including microbial community composition, Operational Taxonomic Unit (OTU) clustering and taxonomic annotation, beta diversity analysis, and Linear Discriminant Analysis Effect Size (LEfSe) analysis.

#### 2.2.3 Statistical methods

Statistical analyses were performed using SPSS for Windows, version 28. A two-sided *p*-value of < 0.05 was considered indicative of statistical significance. Categorical variables were analyzed using the chi-squared test. Continuous variables were assessed using the *t*-test or rank-sum test, as appropriate.

## 3 Results

### 3.1 Community composition analysis

Species composition was analyzed at both the phylum and genus levels across groups, and the results were visualized using pie charts.

*Phylum-level composition*: In Group A (individuals with IC), the predominant bacterial phyla were Proteobacteria (44.41%), Bacteroidetes (30.47%), Firmicutes (17.09%), Verrucomicrobia (6.4%), Actinobacteria (1.01%), and others (0.62%). In Group B (control group), the dominant phyla included Firmicutes (41.55%), Proteobacteria (18.53%), Actinobacteria (13.94%), Bacteroidetes (12.73%), Verrucomicrobia (11.52%), Synergistetes (1.23%), and others (0.5%).

*Genus-level composition*: In Group A the most abundant genera were *Bacteroides* (22.65%), *Halomonas* (17.38%), *Escherichia-Shigella* (7.61%), *Klebsiella* (7.21%), *Akkermansia* (6.4%), *Enhydrobacter* (4.46%), *Prevotella* (3.7%), *Alistipes* (2.4%), *Rhizobium* (2.32%), unclassified *Lachnospira* (1.99%), *Streptococcus* (1.54%), and other genera (21.26%). In Group B, dominant genera included *Akkermansia* (11.52%), *Bacteroides* (8.18%), *Bifidobacterium* (7.79%), *Rothia* (6.36%), *Klebsiella* (6.12%), *Escherichia-Shigella* (5.82%), unclassified *Enterobacter* (4.65%), *Megasphaera* (4.21%), *Collinsella* (3.7%), *Clostridium sensu stricto* (3.68%), *Blautia* (3.27%), *Prevotella* (2.77%), unclassified *Lachnospira* (2.73%), *Subdoligranulum* (2.51%), *Lachnospiraceae/Lachnospira* (2.01%), *Megamonas* (1.48%), *Actinomyces* (1.39%), *Ruminococcus* (1.21%); unclassified *Clostridium* (1.13%), and other genera (19.49%).

**Figure 1:**
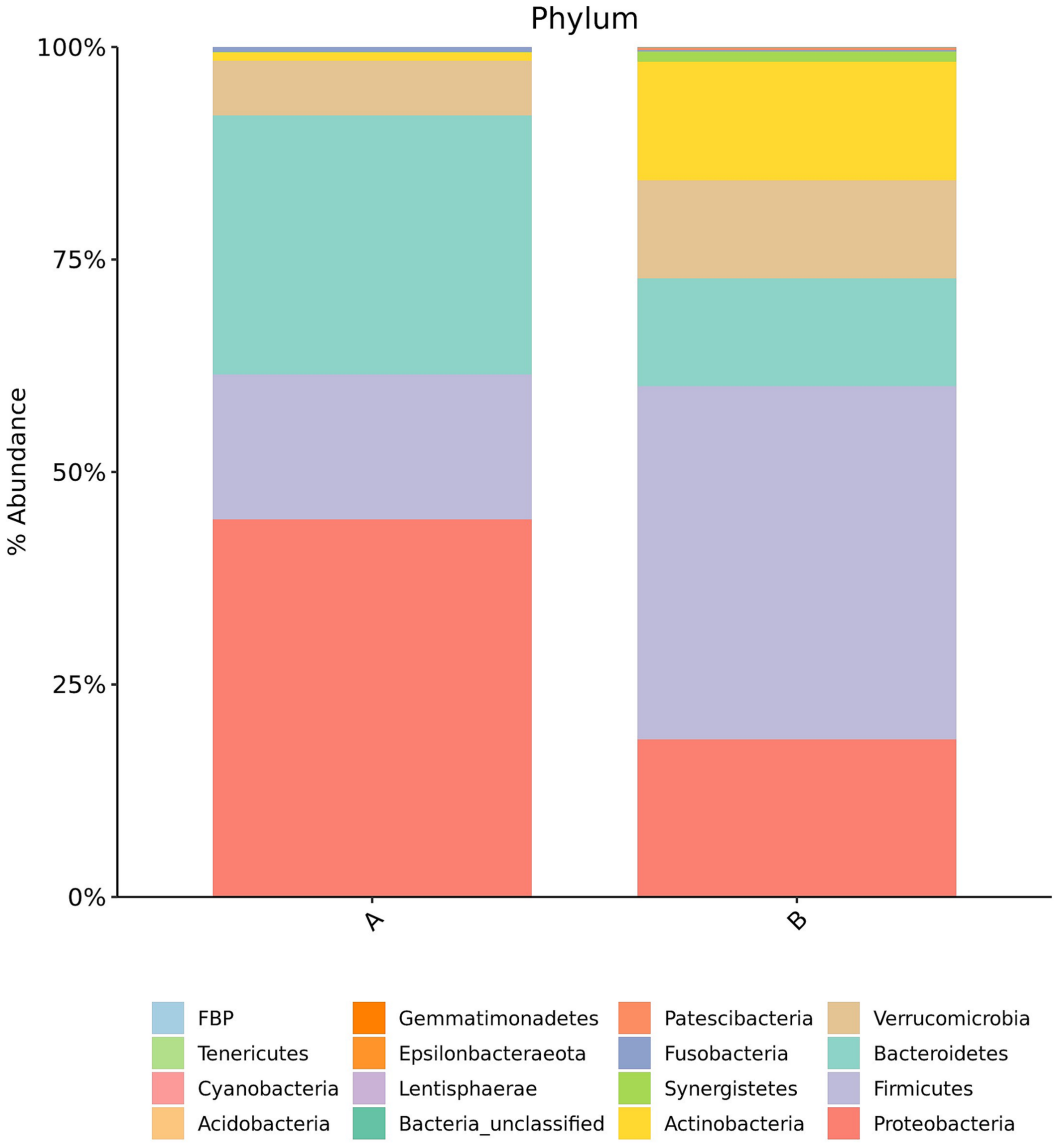
Pie charts showing gut microbiota composition at the phylum level in Group A (IC) and Group B (control). Each color represents a distinct phylum. The relative size of each sector corresponds to the proportional abundance of that taxon within the group.

**Figure 2:**
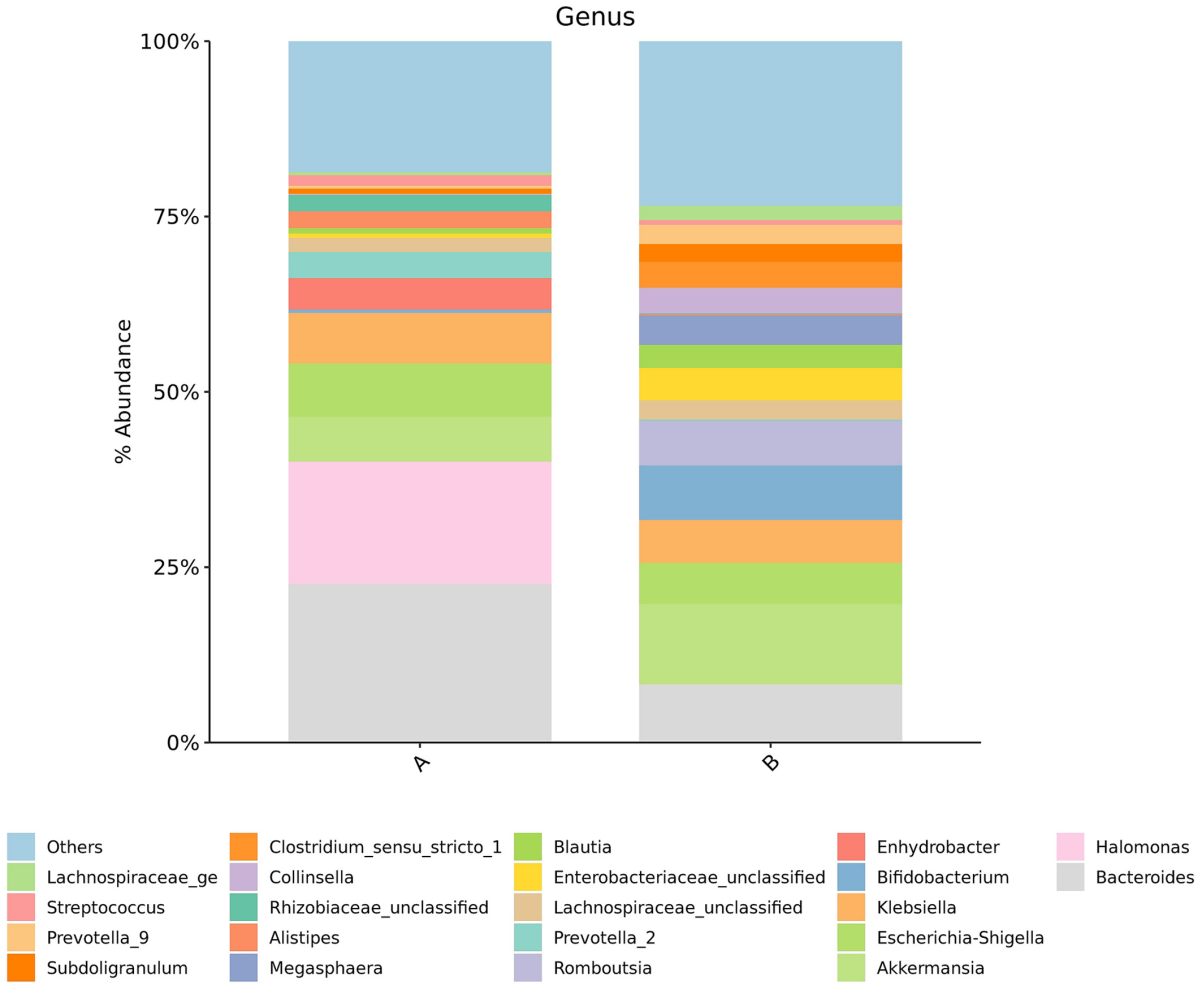
Pie charts showing gut microbiota composition at the genus level in Group A and (IC) and Group B (control). Each color represents a distinct genus. The relative size of each sector reflects the proportional abundance of that taxon within the group.

At the phylum level, individuals in Group A exhibited a relative increase in the abundance of Proteobacteria and Bacteroidetes, accompanied by a reduction in Firmicutes and Actinobacteria (Figure 1). At the genus level, Group A demonstrated increased abundance of *Bacteroides*, *Halomonas*, and *Escherichia-Shigella* while the relative abundance of *Akkermansia* and *Bifidobacterium* were reduced (Figure 2).

### 3.2 Beta diversity analysis

Beta diversity was evaluated to assess differences in microbial community diversity between groups. This analysis included OTU classification, Principal Coordinate Analysis (PCoA), and statistical comparisons of community composition.

#### 3.2.1 OTU classification analysis

**Figure 3:**
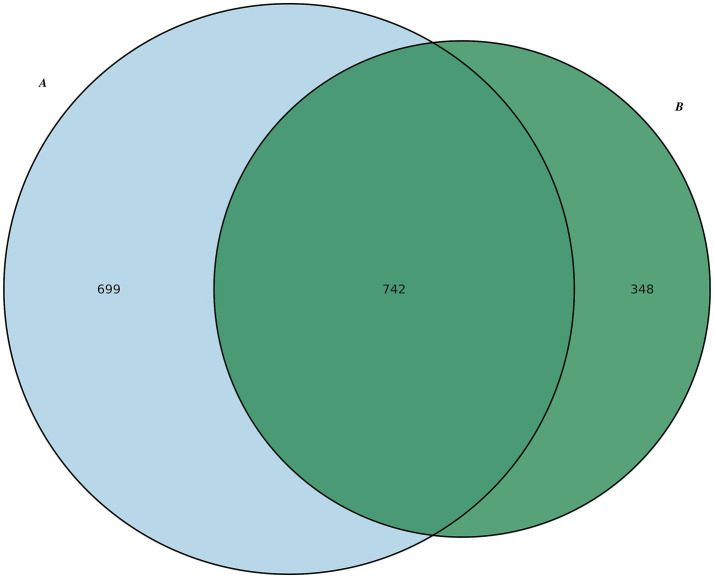
Venn diagram showing the distribution of operational taxonomic units (OTUs) between Group A (IC) and Group B (control). Blue represents Group A and green represents Group B. The numbers indicate the number of OTUs.

A total of 1,469,726 sequences were obtained from Group A and 459,389 sequences from Group B. Sequences sharing ≥ 97% similarity were clustered into the same OTU, representing species-level classification. A total of 1,789 OTUs were identified across both groups. Based on the OTU clustering results, a Venn diagram was generated. Group A exhibited 699 unique OTUs, while Group B contained 348 unique OTUs; 742 OTUs were shared between both groups. These findings indicate that species richness was greater in Group A compared to Group B (Figure 3).

#### 3.2.2 PCoA analysis

**Figure 4:**
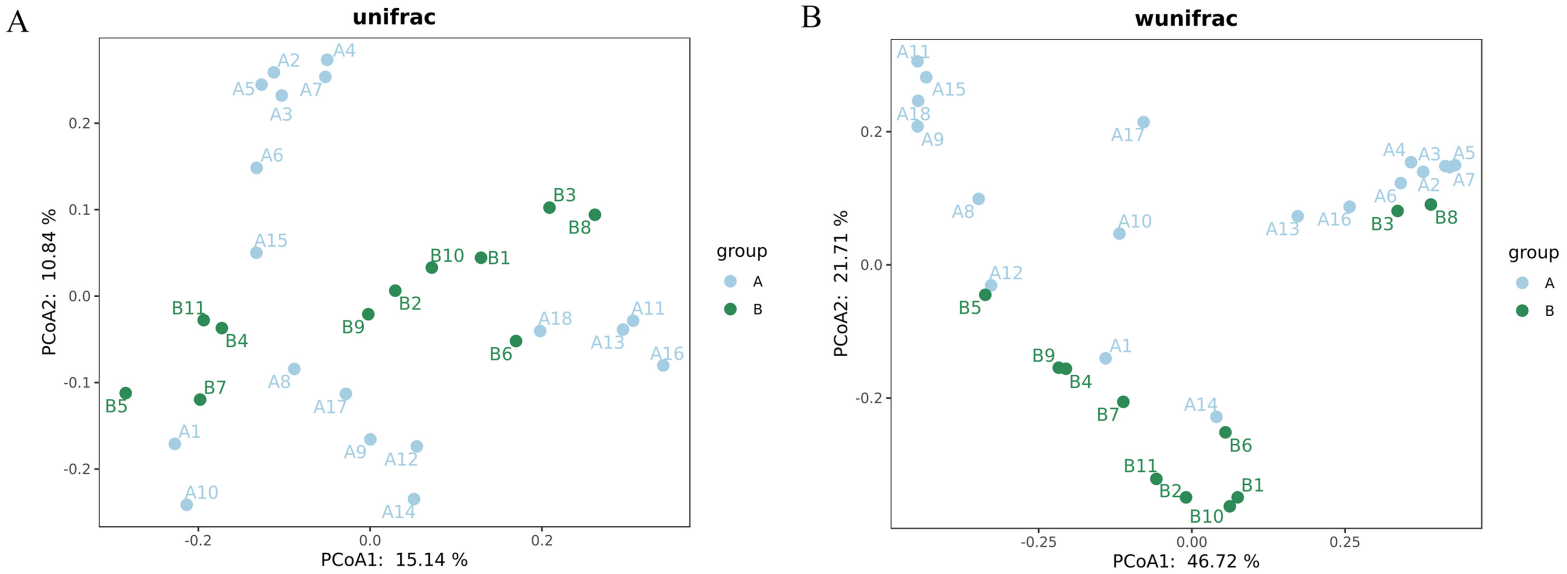
PCoA analysis based on unweighted and weighted UniFrac distance metrics. The *x*-axis represents the first principal coordinate, and the *y*-axis represents the second principal coordinate; the corresponding percentages reflect the proportion of variation explained by each axis. Each point represents an individual sample, with color indicating group membership. Greater distances between points reflect greater differences in microbial community composition. Axis scales are relative and do not represent absolute spatial measurements. Samples clustering closer together indicate higher compositional similarity.

PCoA was performed using UniFrac distance metrics, which incorporate both phylogenetic relationships and community composition. Unweighted UniFrac considers only the presence or absence of taxa, whereas weighted UniFrac takes species abundance into account, offering a more comprehensive evaluation of community differences. For example, if two communities contain the exact same species, the unweighted UniFrac distance would be zero regardless of the differences in abundance. The unweighted UniFrac analysis revealed greater variability in community composition within Group A compared to Group B. Weighted UniFrac analysis confirmed Group A exhibited more pronounced differences in microbial structure relative to Group B. In the resulting PCoA plot, samples from Group A were distributed farther from those of Group B, primarily along the vertical axis, indicating distinct microbial community structures and higher microbial abundance in Group A (Figure 4).

#### 3.2.3 Intergroup community structure difference significance testing

① Analysis of similarities (ANOSIM)

ANOSIM is a non-parametric test primarily used to assess differences in microbial community structure between predefined groups. It assesses whether the dissimilarities between groups are significantly greater than the differences within groups, thereby providing a statistical basis for group differentiation. In ANOSIM, pairwise distances between samples are ranked and categorized as either “between-group” or “within-group” distances. These distributions can be visualized using box plots. Non-overlapping notches in the box plots indicate a statistically significant difference between medians. When the “between-group” distribution is positioned higher along the vertical axis than the “within-group” distributions, it indicates that intergroup variability exceeds intragroup variability.

**Figure 5:**
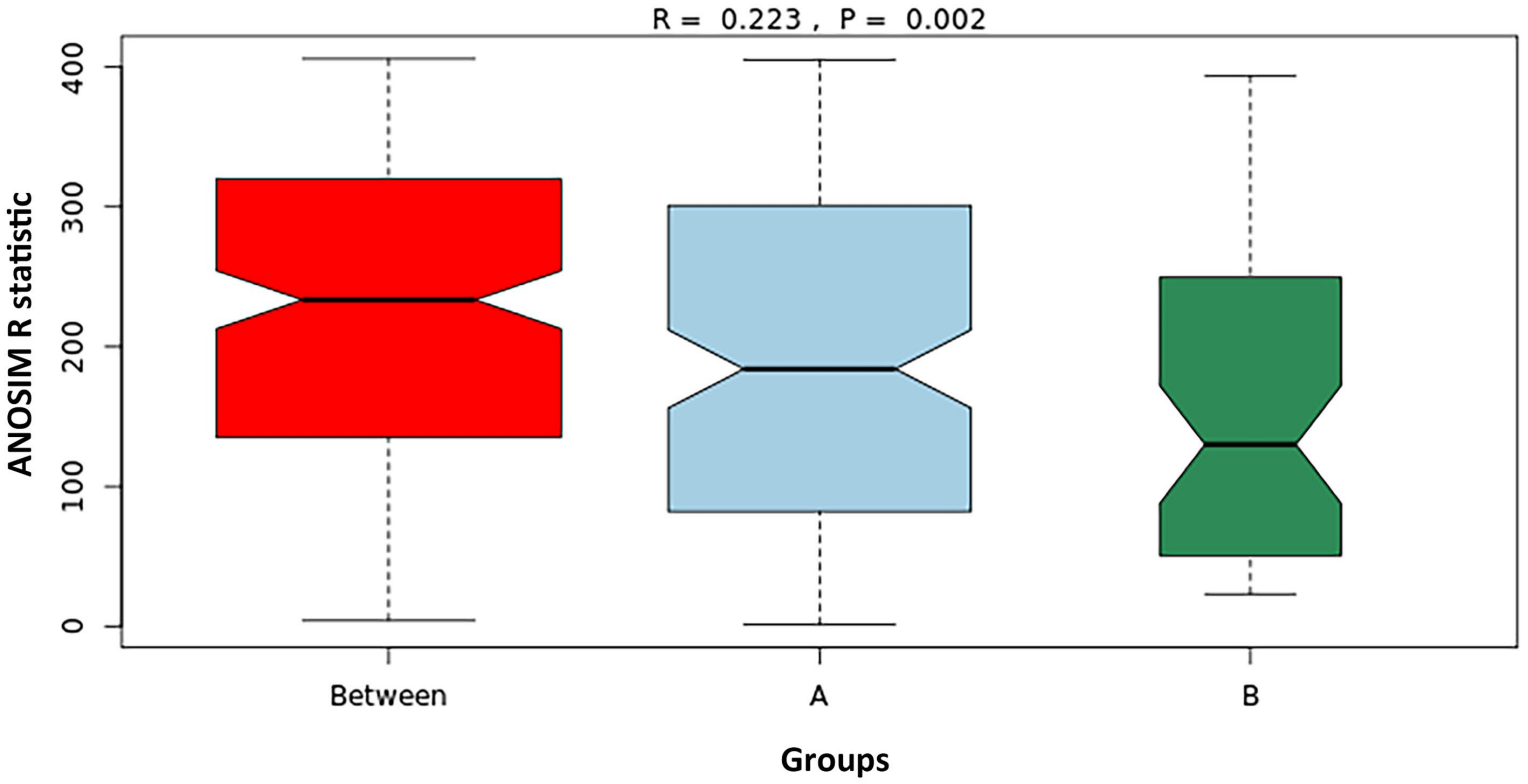
ANOSIM plot comparing microbial community differences between Group A (IC) and Group B (control). “A” and “B” represent within-group variation for each respective group, while “Between” indicates the variation between groups.

The ANOSIM R statistic ranges from −1 to 1. A positive R value (R > 0) indicates greater dissimilarity between groups than within groups, with larger R values reflecting more distinct separation. An R < 0 suggests higher within-group variability. In this study, ANOSIM yielded an R value of 0.223 with a *p* value of 0.002, indicating that microbial community differences between groups were statistically significant and greater than the variation observed within each group (Figure 5). This supports the presence of distinct microbial compositions between Groups A and B.

② Analysis of molecular variance (AMOVA)

AMOVA is a non-parametric statistical approach used to assess the significance of differences in microbial community structure between groups, based on distance matrices such as UniFrac and wUniFrac. In this study the amova function within the Mothur software package was used to analyze intergroup variation. Multiple distance metrics, including Bray-Curtis, Jaccard, UniFrac, and wUniFrac, were applied to compare Groups A and B. Across all metrics, the analysis revealed significant differences in microbial abundance and diversity between the two groups (Table 1).

③ Analysis of PERMANOVA after adjustment for comorbidities

**Table 1:** Comparison of microbial community structure between Group A (IC) and Group B (control) using four molecular variance analysis methods.

Method of calculation	Total variance	Degrees of freedom	Mean variance	*F* value	*p* value
Bray-Crutis	11.5433	28	0.39059	2.55345	0.001001^*^
Jaccard	12.4364	28	0.428281	2.03795	0.003003^*^
UniFrac	5.80214	28	0.196623	2.50894	0.001001^*^
wUniFrac	5.49984	28	0.179959	3.56165	0.013013^*^

*p < 0.05.

Comorbidity was included as variables in the multivariate PERMANOVA model to adjust for confounding effects. To determine the independent effect of disease status on the microbial community structure, we further performed permutational multivariate analysis of variance (PERMANOVA) using multiple distance metrics including Bray-Curtis, Jaccard, unweighted UniFrac, and weighted UniFrac distances, with adjustment for common comorbidities: hypertension, dyslipidemia with fatty liver, atherosclerosis, and diabetes mellitus. After adjustment for these comorbidities, the microbial community structure remained significantly different between Group A and Group B across all distance metrics (Bray-Curtis: *F* = 2.76, R^2^ = 0.082, *p* = 0.006; Jaccard: *F* = 2.69, R^2^ = 0.080, *p* = 0.007; unweighted UniFrac: *F* = 2.71, R^2^ = 0.081, *p* = 0.006; weighted UniFrac: *F* = 2.82, R^2^ = 0.084, *p* = 0.005). In contrast, none of the comorbidities showed a significant independent effect on microbial community structure (all *p* > 0.05). These results indicate that disease status, rather than comorbidities, acts as an independent factor influencing gut microbiota structure.

### 3.3 Differential analysis (LEfSe analysis)

LEfSe is a statistical method designed to identify biological markers, such as taxa that differ significantly in abundance between groups. It combines non-parametric statistical testing with Linear Discriminant Analysis (LDA) to detect potential microbial biomarkers and assess their effect size. In this study, the LDA score threshold was set at ≥2, and no multiple-testing correction was applied. Results are typically visualized through LDA score distribution bar plots and phylogenetic cladograms.

In this study, LEfSe analysis identified distinct taxa that were differentially abundant between Groups A and B. In Group A, the taxa with the highest LDA scores, in descending order, included Proteobacteria, Gammaproteobacteria, Pseudomonadales, Moraxellaceae, *Enhydrobacter*, Eggerthellaceae, Coriobacteriia, Coriobacteriales, Enterococcaceae, and *Enterococcus*.

**Figure 6:**
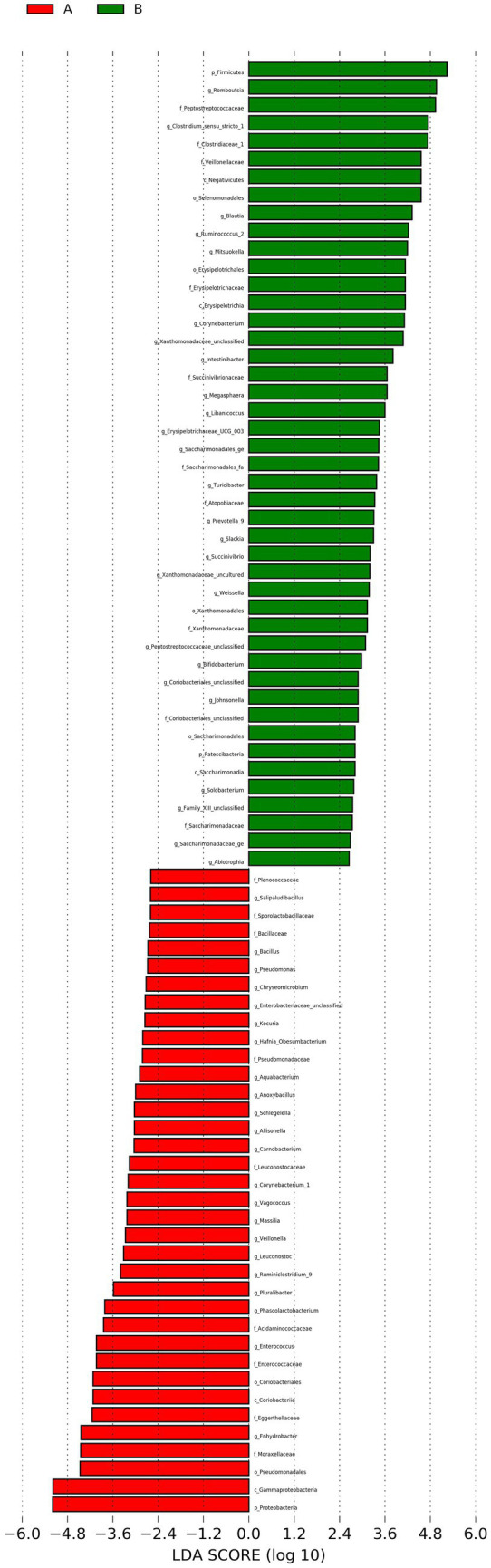
LDA score distribution bar chart. Each bar represents a taxon with an LDA score > 2 and statistically significant differential abundance. Bar length reflects the LDA score, and color indicates the group in which the taxon is more abundant.

In Group B (controls), the highest LDA scores were observed for Firmicutes, *Rothia*, Peptostreptococcaceae, *Clostridium sensu stricto*, Clostridiaceae, Veillonellaceae, Negativicutes, Selenomonadales, *Blautia*, and *Ruminococcus*. These findings indicate significant differences in microbial composition between Groups A and B. The LDA score distribution bar chart is presented in Figure 6.

**Figure 7:**
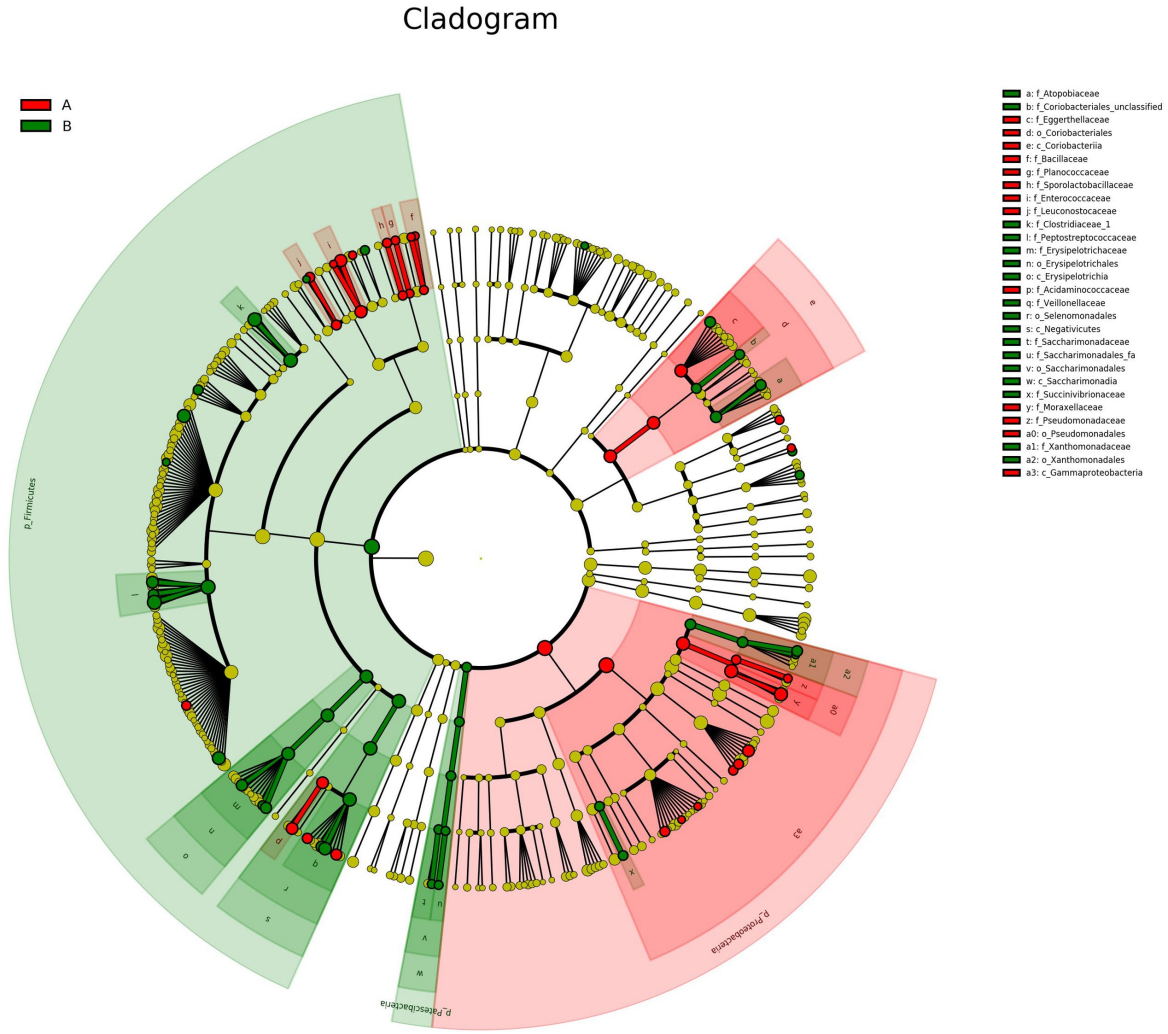
LEfSe cladogram (evolutionary branching diagram). Concentric rings represent taxonomic levels from phylum to genus (or species). Each circle represents a taxon, with circle size proportional to relative abundance. Taxa with no significant differences are shown in yellow. Taxa significantly enriched in each group are colored according to group: red for Group A, and green for Group B. Absence of a group color indicates no significantly enriched taxa in that group.

The LEfSe cladogram is presented in Figure 7. When interpreted with the LDA score distribution bar chart, the results indicate that Group A was predominantly enriched in taxa within the phylum Proteobacteria, whereas Group B showed greater enrichment in taxa belonging to Firmicutes. In Group A, taxa with high LDA scores and widespread phylogenetic differences belonged to Proteobacteria, including Gammaproteobacteria, Pseudomonadales, Coriobacteriales, and Coriobacteriia. Representative genera and families included *Moraxella*, Pseudomonadaceae, Bacillaceae, Acidaminococcaceae, and Leuconostocaceae.

In contrast, Group B showed enrichment of taxa primarily affiliated with Firmicutes including Saccharimonadales, Saccharimonadia, Erysipelotrichia, Erysipelotrichales, Erysipelotrichaceae and Negativicutes. Key enriched taxa included Clostridiaceae, Peptostreptococcaceae, Veillonellaceae, Xanthomonadales, Xanthomonadaceae, Atopobiaceae, and Saccharimonadaceae.

## 4 Discussion

In recent years, the incidence of IC has shown a notable increase, drawing growing attention from the scientific community. However, the underlying pathogenesis of IC remains unclear. While some researchers have proposed a potential association with hypercoagulable states, this hypothesis alone does not fully account for complexity of IC development (Ruan et al., 2020). The gut microbiota, often described as a “functional organ,” due to its extensive role in host physiology and immune modulation, has emerged as a focal point in the study of gastrointestinal diseases. The application of 16S rRNA gene sequencing (V3–V4 region) has enabled comprehensive characterization of microbial communities and elucidation of interspecies relationships, thereby advancing insights into the role of gut microbiota in intestinal inflammation. A growing body of evidence suggests that alterations in microbial composition are associated with inflammatory bowel diseases (IBD).

Prior studies have demonstrated that intestinal levels of potentially pathogenic bacteria such as *E. coli*, *Enterococcus*, and *Bacteroides* are elevated in individuals with IBD, while beneficial gut taxa such as *Lactobacillus*, *Bifidobacterium*, and *Clostridium leptum* are comparatively depleted (Wang et al., 2018). Additionally, decreased microbial diversity and increased microbial relative abundance of *Proteobacteria* have been frequently reported in IBD cohorts (Santoru et al., 2017).

In a previous study conducted by our team on the gut microbiota of individuals with Crohn’s disease, elevated levels of Coriobacteriaceae, Alcaligenaceae, Erysipelotrichaceae, and Streptococcaceae were observed in affected individuals compared to healthy controls (Shen et al., 2016). In contrast, the relative abundance of Prevotellaceae, Bacteroidaceae, and Bifidobacterium was significantly reduced.

Currently, few studies have explored the relationship between IC and the gut microbiota. The fundamental pathophysiological mechanism of IC is generally attributed to ischemia–reperfusion injury of the intestinal mucosa (Moszkowicz et al., 2013). Emerging evidence suggests that the gut microbiota may confer protection against ischemia/reperfusion-induced intestinal damage through nucleotide-binding oligomerization domain-containing protein 2 (NOD2) signaling. NOD2 functions as a pattern recognition receptor that detects muramyl dipeptide, a conserved component of the peptidoglycan layer found in both Gram-positive and Gram-negative bacterial cell walls.

In preclinical models of necrotizing enterocolitis, activation of the NOD2 signaling pathway has been shown to mitigate hypoxia-induced intestinal injury, partly by downregulating Toll-like receptor 4 (TLR4)–mediated inflammatory responses (Tatum et al., 2010). These findings suggest that the downregulation of NOD2 signaling, potentially driven by gut microbiota dysbiosis, may be associated with the pathophysiological process of IC, although this inference requires further experimental validation.

Previous studies have reported that mucosal biopsies from both inflamed and non-inflamed regions in individuals with IBD, compared with those from healthy controls, exhibit a decreased abundance of Firmicutes and an increased abundance of Bacteroidetes (Walker et al., 2011). These findings suggest that alterations in gut microbial composition following the onset of IC may contribute to intestinal inflammation. Although dysbiosis can result from multiple host or environmental factors, an increased relative abundance of Proteobacteria has been frequently identified as a hallmark of microbial imbalance (Shin et al., 2015; Rusconi et al., 2017). This observation is consistent with the present study’s findings.

Rusconi et al. (2017) proposed that dysbiotic colonic microbiota could serve as a biomarker for necrotizing colitis, characterized by reduced microbial abundance and diversity, increased abundance of Proteobacteria, and decreased abundance of Firmicutes and Bacteroidetes. However, findings related to Bacteroidetes have varied across studies. In the current study, an increased abundance of both the Bacteroidetes and the *Bacteroides* was observed in individuals with IC, suggesting that these taxa may contribute to the pathophysiological mechanisms underlying IC.

A previous study by Dahal et al. (2023) examined gut microbiota composition in four individuals with IC and compared the findings with those from 10 healthy controls. The study reported reduced species richness in the IC group, along with compositional imbalances at multiple taxonomic levels, including phylum, order, family, genus, and species. Notably, the abundances of *Enterococcus faecium*, *E. coli*, and *Enterococcus faecalis* were significantly elevated in individuals with IC and were proposed as potential microbial biomarkers. In contrast to the findings of Dahal et al. (2023), the present study identified a higher number of OTUs in the IC group compared to the control group, indicating greater species richness. Additionally, the PCoA revealed broader dispersion of data points among IC samples relative to controls, suggesting increased microbial divergence and higher overall community diversity in the IC group.

These findings were further supported by intergroup statistical analyses, including ANOSIM and AMOVA, which demonstrated significant differences in microbial composition, with the IC group exhibiting higher diversity. It should be clarified that higher microbial diversity does not necessarily mean a healthier microbiota. The health status of the gut microbiota depends on the stability of the community structure, the ratio of beneficial bacteria to pathogenic bacteria, and functional synergy, rather than mere species richness. The high diversity in IC patients in this study may reflect the instability of the microbial community: ischemic injury leads to damage of the intestinal mucosal barrier, providing conditions for the invasion and colonization of opportunistic pathogens (such as *Escherichia-Shigella*, Halomonas, etc.). The addition of these foreign taxa increases species richness but disrupts the balance of the original microbiota. This “increased diversity” is more likely a manifestation of microbial dysbiosis rather than an indication of community resilience. It is essentially an imbalance in community structure caused by the decreased abundance of beneficial bacteria (such as Bifidobacterium, Rothia) and the invasion of pathogenic bacteria and opportunistic pathogens.

Furthermore, it is important to acknowledge the risk of false positives in small cohorts. This study included only 18 IC patients and 11 healthy controls, with a relatively small total sample size. In microbiome research, small sample sizes may lead to overestimation of the significance of microbial differences or misidentification of spurious associations due to insufficient statistical power and potential sampling bias. The observed increase in microbial diversity in the IC group should therefore be interpreted with caution, as it may partially be attributed to false positive signals arising from the small cohort size. Future studies with larger sample sizes and multicenter validation are needed to confirm the reliability of this finding.

Alterations in microbial diversity among individuals with IC may also be influenced by factors such as chronic constipation, lipid metabolism disorders, and persistent low-grade intestinal inflammation. IC predominantly affects individuals over the age of 65 and is more prevalent in females than males (Yngvadottir et al., 2017). With advancing age, the ratio of Firmicutes to Bacteroidetes has been shown to decrease (Walker et al., 2011). Firmicutes are capable of converting lactic acid into butyrate, a short-chain fatty acid with anti-inflammatory properties, whereas Bacteroidetes produce acetate and propionate, which also contribute to intestinal homeostasis. These findings suggest that reduced abundance of Firmicutes, and consequent potential reductions in butyrate production, may be associated with increased intestinal inflammation and could be involved in the pathophysiological process of IC, although this inference requires verification through direct functional experiments. At the phylum level, Proteobacteria and Bacteroidetes were relatively more abundant in the IC group. Proteobacteria encompasses a range of pathogenic species, including *Salmonella*, *E. coli*, and *Vibrio cholerae*. Previous studies have implicated *Campylobacter*, *Shigella, Salmonella, E. coli* O157: H7, and hemolytic *Proteus* species as potential etiological agents in IC (Washington and Carmichael, 2012). Invasive *E. coli* strains, in particular, can proliferate extensively, disrupt gut microbial diversity and intestinal barrier function, and promote the expression of inflammatory genes, thereby triggering mucosal inflammation (Ahmed et al., 2016).

At the genus level, the IC group exhibited increased abundance of *Bacteroides*, *Escherichia-Shigella* and *Streptococcus*. In contrast, beneficial genera such as *Bifidobacterium* and *Rothia*, both members of Actinobacteria, were reduced. The increased presence of *Escherichia-Shigella* and *Streptococcus* may contribute to the inflammatory milieu observed in IC. *Escherichia-Shigella* (STEC), also referred to as enterohemorrhagic *E. coli* (EHEC), is a Gram-negative, anaerobic bacillus known to cause recurrent hemorrhagic diarrhea, abdominal pain, and systemic symptoms such as fever, nausea, and vomiting. The clinical presentation associated with STEC infection closely resembles the symptomatology of IC, further supporting its potential involvement in disease pathogenesis (Kolodziejek et al., 2022).

*Bifidobacterium* is a well-established probiotic extensively used in clinical practice. It produces short-chain fatty acids such as acetate and valerate. Elevated levels of acetate have been shown to enhance intestinal motility, suggesting a link between reduced levels of *Bifidobacterium* abundance and constipation, one of the known risk factors for IC (El-Salhy et al., 2017). The observed reduction in *Bifidobacterium* in individuals with IC may therefore contribute to disease onset via impaired intestinal transit.

*Roseburia* is another beneficial genus implicated in the regulation of gut microbial diversity. It has been associated with improved glucose metabolism and weight reduction (Seo et al., 2020). LEfSe analysis in the present study indicated that Group A was enriched in several taxa with high LDA scores, including Proteobacteria, Pseudomonadales, Moraxellaceae, *Enhydrobacter*, Coriobacteriia and Coriobacteriales, and *Enterococcus*, all of which demonstrated statistically significant differences in abundance. In contrast, the control group was enriched in Firmicutes, *Rothia*, and Erysipelotrichales/Erysipelotrichaceae.

Previous studies have indicated that increased abundance of *Erysipelothrix* and *Rhizobia* is negatively correlated with intestinal inflammation, suggesting a potential probiotic role for these genera (Sato et al., 2021). However, the specific mechanisms underlying their anti-inflammatory effects remain unclear and warrant further investigation. Overall, the maintenance of intestinal mucosal barrier integrity is essential for preserving gut microbiota homeostasis. A stable barrier supports resistance to overgrowth of potentially pathogenic microorganisms and prevents bacterial translocation and toxin dissemination. These microbial community changes are associated with IC, and they may affect the pathological process of IC by disrupting intestinal homeostasis, promoting inflammatory responses, and impairing mucosal barrier function, but the specific mechanisms require further clarification through prospective studies and mechanism validation experiments.

It should be noted that there are comorbidities such as hypertension (11 cases), dyslipidemia and fatty liver (9 cases), atherosclerosis (7 cases), and diabetes mellitus (1 case) in the IC group. Existing studies have shown that these diseases can affect the composition of the gut microbiota independently of IC. For example, the abundance of Proteobacteria may be increased in hypertensive patients, and the abundance of Bifidobacterium may be decreased in diabetic patients. Although this study controlled for some confounding factors through baseline characteristic matching, these comorbidities may still have an additive effect on the gut microbiota structure of IC patients, and their interference as confounding factors on the study results cannot be completely excluded.

This study has certain limitations: Firstly, the sample size between the IC group and the control group did not reach a 1:1 balance, and the sample size of the control group was relatively small, which may affect the statistical test power and increase the risk of potential bias; future studies can expand the sample size and adopt a more rigorous 1:1 matching design to improve the reliability of the results. Secondly, the IC group subjects had multiple comorbidities, which may independently affect the gut microbiota, increasing the complexity of result interpretation and bringing challenges to confounding factor control; future studies can use stratified analysis or multivariate regression models to further adjust the potential impact of comorbidities. In addition, this study did not directly evaluate dietary and environmental factors (such as dietary fiber intake, living habits, etc.). These factors are important regulatory factors of the gut microbiota, but due to the lack of direct detection data, their impact cannot be quantified; the impact of dietary factors is no longer used as an explanatory factor for the differences in the microbiota of IC patients in this paper, which is an important limitation of this study. Future studies can supplement relevant data through dietary questionnaires and lifestyle surveys to more comprehensively analyze the driving factors of microbiota changes. It should be noted that OTU-based clustering has inherent limitations, such as arbitrary similarity thresholds that may obscure fine-scale microbial diversity, and ASV-based approaches are recommended for future high-resolution studies.

## 5 Conclusion

Individuals with IC demonstrate increased gut microbiota diversity; however notable alterations in community composition are evident. At the phylum level, the relative abundance of Proteobacteria and Bacteroidetes is elevated, while Firmicutes and Actinobacteria are markedly reduced in individuals with IC. At lower taxonomic ranks, increased relative abundance is observed for the order Pseudomonadales, the family Coriobacteridae, and the genera *Bacteroides*, *Halomonas*, and *Escherichia-Shigella*. Conversely, beneficial genera such as *Akkermansia*, *Bifidobacterium*, and *Rothia* are significantly diminished. These microbial shifts are associated with IC, which may be related to the disruption of gut homeostasis, promotion of inflammation, and impairment of mucosal barrier function. Due to the observational and cross-sectional nature of this study, no causal relationship between microbial changes and IC can be established, and further research is needed to explore the specific mechanisms involved.

## Data Availability

The datasets generated and/or analyzed during the current study are available in the NGDC Genome Sequence Archive (GSA) (CRA03039).

## References

[ref1] Ahmed I. Roy B. C. Khan S. A. Septer S. Umar S. (2016). Microbiome, metabolome and inflammatory bowel disease. Microorganisms 4:20. doi: 10.3390/microorganisms4020020, 27681914 PMC5029486

[ref2] Dahal R. H. Kim S. Kim Y. K. Kim E. S. Kim J. (2023). Insight into gut dysbiosis of patients with inflammatory bowel disease and ischemic colitis. Front. Microbiol. 14:1174832. doi: 10.3389/fmicb.2023.1174832, 37250025 PMC10211348

[ref3] Editorial Board of Chinese Journal of Geriatric Medicine (2011). Chinese experts’ recommendations on diagnosis and treatment of ischemic bowel disease in the elderly (2011). J. Geriatr. 30, 1–6. doi: 10.3760/cma.j.issn.0254-9026.2011.01.001

[ref4] El-Salhy M. Ystad S. O. Mazzawi T. Gundersen D. (2017). Dietary fiber in irritable bowel syndrome (review). Int. J. Mol. Med. 40, 607–613. doi: 10.3892/ijmm.2017.3072 28731144, 28731144 PMC5548066

[ref5] Kolodziejek A. M. Minnich S. A. Hovde C. J. (2022). *Escherichia coli* 0157:H7 virulence factors and the ruminant reservoir. Curr. Opin. Infect. Dis. 35, 205–214. doi: 10.1097/QCO.0000000000000834, 35665714 PMC9302714

[ref6] Moszkowicz D. Mariani A. Trésallet C. Menegaux F. (2013). Ischemic colitis: the ABCs of diagnosis and surgical management. J. Visc. Surg. 150, 19–28. doi: 10.1016/j.jviscsurg.2013.01.002, 23433833

[ref8] Ruan S. L. Gu X. J. Guan Q. B. (2015). Domestic literature survey on the epidemiology and clinical features of ischemic colitis. Chin. J. Geriatr. 34, 565–569. doi: 10.3760/cma.j.issn.0254-9026.2015.05.029

[ref9] Ruan S. L. Wang W. G. Zhou X. F. Du Y. Y. Shen H. Y. (2020). The role of hypercoagulability and platelet activation in the pathogenic mechanism of ischemic colitis. Jiangsu Med. J. 46, 40–42. doi: 10.19460/j.cnki.0253-3685.2020.01.011

[ref10] Rusconi B. Good M. Warner B. B. (2017). The microbiome and biomarkers for necrotizing enterocolitis: are we any closer to prediction? J. Pediatr. 189, 40–47.e2. doi: 10.1016/j.jpeds.2017.05.075, 28669607 PMC5614810

[ref11] Santoru M. L. Piras C. Murgia A. Palmas V. Camboni T. Liggi S. . (2017). Cross sectional evaluation of the gut-microbiome metabolome axis in an Italian cohort of IBD patient. Sci. Rep. 7:9523. doi: 10.1038/s41598-017-10034-5 28842640 PMC5573342

[ref12] Sato Y. Atarashi K. Plichta D. R. Arai Y. Sasajima S. Kearney S. M. . (2021). Novel bile acid biosynthetic pathways are enriched in the microbiome of centenarians. Nature 599, 458–464. doi: 10.1038/s41586-021-03832-5, 34325466

[ref13] Seo B. Jeon K. Moon S. Lee K. Kim W. K. Jeong H. . (2020). Roseburia spp. abundance associates with alcohol consumption in humans and its administration ameliorates alcoholic fatty liver in mice. Cell Host Microbe 27, 25–40.e6. doi: 10.1016/j.chom.2019.11.001, 31866426

[ref14] Shen H. Y. Ruan S. L. Xu S. L. Lu Q. M. Yang Z. H. Chen C. X. . (2016). Alterations of bacterial diversity of intestinal microbiota in patients with Crohn’s diseases. Chin. J. Microecol. 28, 662–666. doi: 10.13381/j.cnki.cjm.201606008

[ref15] Shin N. R. Whon T. W. Bae J. W. (2015). Proteobacteria: microbial signature of dysbiosis in gut microbiota. Trends Biotechnol. 33, 496–503. doi: 10.1016/j.tibtech.2015.06.011, 26210164

[ref16] Tatum P. M. Harmon C. M. Lorenz R. G. Dimmitt R. A. (2010). Toll-like receptor 4 is protective against neonatal murine ischemia-reperfusion intestinal injury. J. Pediatr. Surg. 45, 1246–1255. doi: 10.1016/j.jpedsurg.2010.02.093, 20620328 PMC2952414

[ref17] Walker A. W. Sanderson J. D. Churcher C. Parkes G. C. Hudspith B. N. Rayment N. . (2011). High-throughput clone library analysis of the mucosa-associated microbiota reveals dysbiosis and differences between inflamed and non-inflamed regions of the intestine in inflammatory bowel disease. BMC Microbiol. 11:7. doi: 10.1186/1471-2180-11-7, 21219646 PMC3032643

[ref18] Wang Z. W. He Y. Zeng N. Y. Tang W. Zhou H. (2018). Regional variation and diagnosis modeling of gut microbiome for inflammatory bowel diseases. Chin. J. Lab. Med. 41, 734–741. doi: 10.3760/cma.j.issn.1009-9158.2018.10.007

[ref19] Washington C. Carmichael J. C. (2012). Management of Ischemic Colitis. Clin. Colon Rectal Surg. 25, 228–235. doi: 10.1055/s-0032-1329534, 24294125 PMC3577613

[ref20] Yngvadottir Y. Karlsdottir B. R. Hreinsson J. P. Ragnarsson G. Mitev R. U. M. Jonasson J. G. . (2017). The incidence and outcome of ischemic colitis in a population-based setting. Scand. J. Gastroenterol. 52, 704–710. doi: 10.1080/00365521.2017.1291718, 28276832

